# Chemotherapy followed by low dose radiotherapy in childhood Hodgkin's disease: retrospective analysis of results and prognostic factors

**DOI:** 10.1186/1748-717X-1-38

**Published:** 2006-10-02

**Authors:** Gustavo A Viani, Marcus S Castilho, Paulo E Novaes, Celia G Antonelli, Robson Ferrigno, Cassio A Pellizzon, Ricardo C Fogaroli, Maria A Conte, Joao V Salvajoli

**Affiliations:** 1 Department of Radiation Oncology Hospital do Câncer, SaoPaulo, Brazil; 2 Department of Pediatric Oncology Hospital do Câncer, Sao Paulo, Brazil

## Abstract

**Purpose:**

To report the treatment results and prognostic factors of childhood patients with Hodgkin's disease treated with chemotherapy (CT) followed by low dose radiotherapy (RT).

**Patients and methods:**

This retrospective series analyzed 166 patients under 18 years old, treated from January 1985 to December 2003. Median age was 10 years (range 2–18). The male to female ratio was 2,3 : 1. Lymphonode enlargement was the most frequent clinical manifestation (68%), and the time of symptom duration was less than 6 months in 55% of the patients. In histological analysis Nodular Sclerosis was the most prevalent type (48%) followed by Mixed Celularity (34.6%). The staging group according Ann Arbor classification was: I (11.7%), II (36.4%), III (32.1%) and IV (19.8%). The standard treatment consisted of chemotherapy multiple drug combination according the period of treatment protocols vigent: ABVD in 39% (n-65) of the cases, by VEEP in 13 %(n-22), MOPP in 13 %(n-22), OPPA-13 %(n-22) and ABVD/OPPA in 22 %(n-33). Radiotherapy was device to all areas of initial presentation of disease. Dose less or equal than 21 Gy was used in 90.2% of patients with most part of them (90%) by involved field (IFRT) or mantle field.

**Results:**

The OS and EFS in 10 years were 89% and 87%. Survival according to clinical stage as 94.7%, 91.3%, 82.3% and 71% for stages I to IV(p = 0,005). The OS was in 91.3% of patients who received RT and in 72.6% of patients who did not (p = 0,003). Multivariate analysis showed presence of B symptoms, no radiotherapy and advanced clinical stage to be associated with a worse prognosis.

**Conclusion:**

This data demonstrating the importance of RT consolidation with low dose and reduced volume, in all clinical stage of childhood HD, producing satisfactory ten years OS and EFS. As the disease is highly curable, any data of long term follow-up should be presented in order to better direct therapy, and to identify groups of patients who would not benefit from radiation treatment.

## Background

During the last two decades, treatment strategies of pediatric Hodgkin's disease have changed considerably. Radiotherapy was the standard treatment for limited (stage I or II) aggressive lymphoma until 1980, although the five-year rate of disease-free survival was less than 50 percent [1]. Subsequently, chemotherapy was added to involved-field radiotherapy (chemo radiotherapy) with the goals of controlling occult systemic disease and reducing the size of irradiation fields [2]. Other investigators, however, showed that chemotherapy alone could cure limited stage lymphoma [3]; as a result, either chemo radiotherapy or chemotherapy alone was used for localized disease [4,5].

The vast majority of children with Hodgkin's disease nowadays have an excellent chance of definite cure [1]. Therefore the main goal of Hodgkin's disease studies has been to minimize long-term side effects while maintaining the high cure rates. Combined chemotherapy and radiotherapy regimens have become the standard approach because they allow one to reduce toxicity while maintaining high overall efficacy. In these regimens, chemotherapy as well as radiotherapy is adapted according to stage, spread and volume of disease [6-13]. Most treatment programs for childhood HD consist of combined-modality therapy, with a focus on reducing the dose and field of radiation therapy (RT) and the cumulative doses of cytotoxic agents [4]. Risk-adapted regimens seek to maintain disease control while reducing therapy-related complications. Therefore, this approach may reduce therapy for patients with favourable diagnostic features or intensify therapy for patients with unfavourable disease presentations. Numerous investigations have established that children and adolescents with favourable presentations of Hodgkin's disease are excellent candidates for reduced therapy. Outcomes for unfavourable patients treated with contemporary combined-modality or chemotherapy-alone regimens demonstrate 5-year disease control in the range of 70% to 90 % [10-13]. In this study we analyze a single institution's experience of 15 years on the treatment of childhood Hodgkin's disease.

## Methods

Between January 1985 and December 2000 166 patients younger than 18 years of age were enrolled in this study. Eligible patients had histologically confirmed Hodgkin's disease. Patients with unfavourable disease included all those with Ann Arbor stage III and IV disease, as well as patients with stage I and II disease who had bulky mediastinal lymphadenopathy (defined as the ratio of mediastinal mass to intra-thoracic cavity of one third or greater on upright chest radiograph, or a peripheral lymph node mass greater than 6 cm in largest diameter), or the presence of B symptoms. Clinical staging evaluation included history and physical examination; CBC with differential, erythrocyte sedimentation rate, routine renal and hepatic functions and analysis chemistries; chest radiograph, cervical, abdominal, pelvis and thoracic computed tomography (CT) scan with contrast and bone marrow biopsy. All patients included in this sample were submitted to chemotherapy. The chemotherapy protocols were modified according to the institution's current treatment policy during the time of this study. The chemotherapy protocols consisted predominantly of MOPP or ABVD in the period from 1985 – 1990, ABVD or VEEP between 1990 – 1995 and in the last period ABVD and OPPA + ABVD. Gallium scan and bone scans were also used to monitor response. Radiotherapy was restricted to involved fields (IFRT) in a modified technique in most part of the cases (clinical stage I-II). The patients with advanced clinical stage (III – IV) and some patients with stage II (Bulky disease) were submitted to the upper mantle field. In some cases as clinical stage IV additionally to spleen and para-aortic lymph nodes received radiation. Visceral sites of involvement and bone lesions were treated with different doses according the organ tolerance. Dose per fraction was 1.8 Gy or 2 Gy, for large volumes in small children 1.5 Gy, using five sessions per week with final dose between 20 – 30 Gy in most of cases.

After completion of therapy, patients were followed regularly with physical examination, chest x-ray, and routine laboratory studies. In addition, restaging studies including CT scans of neck, chest, abdomen, and pelvis, and gallium were recommended at 1 and 2 years off therapy. Additional exams were performed as guided by patients' symptoms.

### Study end points

This study retrospectively analysed 15 years of experience in the treatment of Hodgkin's disease in childhood. The primary end point was to evaluate prognosis associated with the use of radiotherapy on combined treatment and the real benefit of adjuvant low dose RT in advanced stages. The secondary objective was to analyse the prognostic factors related to survival (OS) and event free survival (EFS). The prognostic factors analyzed were: age, clinical stage, B symptoms, dose radiotherapy, delivery radiotherapy, chemotherapy, sex and histological subtype.

### Statistical analysis

EFS were defined as the interval from start treatment to the date of first event (relapse or progression, second malignancy, or death from any cause) or to the date of last follow-up. Survival was defined as the interval from start treatment to the date of death from any cause or to last follow-up. EFS and Overall Survival (OS) distributions were estimated using the Kaplan and Meier method. The log-rank test was used to examine differences in EFS, OS and Cox Regression Test for multivariate analysis of significant factors. (p < 0.05)

### Characteristics of patients and treatment

Demographic characteristics of the patient cohort are shown in Table 1. The median age was 10 years (range 2.2 – 18 years). The majority of patients were white (n-138, 83.1%); 69.9% were male (n-116). Clinical Stage distribution was I in 19 patients (11.4%), II in 60 patients (36.1%), III in 55 patients (33.1%), and IV in 32 patients (19.1%). B symptoms were present in 112 patients (67.5%). The median symptoms time until diagnosis was 6 months, the most common manifestation was cervical lymph node enlargement, seen in 71%. The histology was nodular sclerosis 48.1%, followed by mixed celularity 34.3%, lymphocyte depletion 1.8 % and lymphocyte predominant 15.6%. Radiotherapy was delivered to 146 patients (88%). Treatment volumes included mantle field (34.9%), involved field (43.3%), and para-aortic (5.4%), or iliac inguinal femoral lymphatics (4.2%). The median radiation therapy dose was 21 Gy in 1.7 Gy (range 10 – 40 Gy) and distribution of patients submitted to radiotherapy by dose level was: 10–20 Gy (1.3%), 21 – 30 Gy (98 %) and 31 – 40 Gy (0.7%), as showed in table-2. The chemotherapy protocols were modified according to the institution's current treatment policy during time. The children were treated with ABVD in 39% (n-65) of the cases, by VEEP in 13 %(n-22), MOPP in 13 %(n-22), OPPA-13 %(n-22) and ABVD/OPPA in 22 %(n-33). The distribution of fields radiotherapy, doses radiotherapy and chemotherapy protocols used in treatment of patients according to clinical stage is displayed in table-3. All the patients with initial clinical stage (I – II) received radiotherapy with dose less or equal than 21 Gy. Involved field and mantle field radiotherapy was delivery to 72/146 (43.3%) and 58/146 (35%) of patients, respectively. Involved field radiotherapy was used in 62/75 (82.4%) of initial clinical stage (I-II) patients as showed in table-3.

**Table 1:** Patients characteristics

**Age**	**Median (Y)**	**Range**
	10	2.2 – 18
**Sex**	**Male (%)**	**Female (%)**
	116 (69.9)	50 (30.1)
		
**Race**	**White (%)**	**Black (%)**
	138(83.1)	28 (16.9)
		
**Symptoms time**	**Median (M)**	**Range (M)**
	6	1–12
		
**Clinical stage**	**Number(%)**	
I	19 (11.7)	
II	60 (36.1)	
III	55 (33.1)	
IV	32 (19.1)	
		
**Histology**	**Number (%)**	
Sclerosis nodular	80(48.1)	
Mixed celullarity	57(34.3)	
Lymphocitic depletion	26(15.6)	
Predominant lymphocitic	3 (1.8)	
		
**Symptoms B**	**Number (%)**	
Present	112 (67.5%)	
Absent	54 (32.5%)	

**Table 2:** characteristics of treatment

**Radiotherapy**	**Number (%)**	
	146 (88)	
**RT/clinical stage**		
I	17 (10.2)	
II	58 (34,9)	
III	45 (27,1)	
IV	26 (15,6)	
		
**RT dose**	**Median (Gy)**	**Range (Gy)**
	21	10–40
		
**RT/level dose Gy**	**Number (%)**	
10 – 20 Gy	2 (1.3)	
21 – 30 Gy	143 (98)	
31 – 40 Gy	1 (0.7)	
		
**RT field**	**Number (%)**	
Involved field	72 (43,3)	
Mantle field	58 (34,9)	
Infradiafragmatic field	9 (5,4)	
Total nodal irradiation	7 (4,2)	
		
**Chemotherapy**	**Number (%)**	
ABVD	65 (39)	
VEEP	22 (13)	
MOPP	22 (13)	
OPPA	33 (22)	
OPPA +ABVD	22 (13)	

**Table 3:** Radiotherapy Dose, Fields and chemotherapy according clinical stage.

**Radiotherapy**	146 (88)			
	**Clinical**		**Stage**	
**RT/clinical stage**	I	II	III	IV
	17	58	45	26
				
**Dose RT Gy**	I	II	III	IV
< = 21 Gy	17	58	44	26
> 21 Gy	0	0	**1	0
				
**RT field**	I	II	III	IV
Involved field	17	45	8	2
Mantle field	0	13	35	10
Infradiafragmatic field	0	0	2	7
Total nodal irradiation	0	0	0	7
				
**Chemotherapy**	I	II	III	IV
ABVD	17	45	1	0
VEEP	0	13	9	0
OPPA	2*	2*	16	2
MOPP	0	5*	17	0
ABVD/OPPA	0	5*	2	30

*patients submitted to chemotherapy alone

** patients received 40 Gy

## Results

### Overall survival and event free survival rates

**Figure 1:**
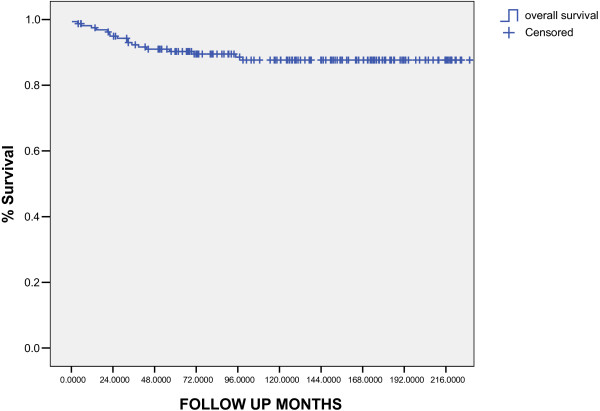
overall survival (Kaplan Meier estimative).

**Figure 2:**
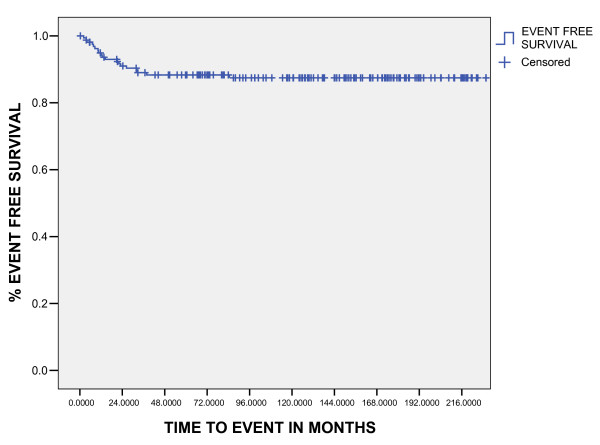
Event Free Survival (Kaplan Meier estimative).

**Figure 6:**
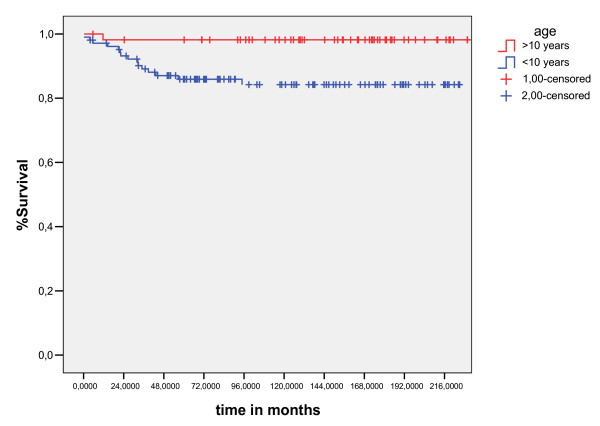
overall survival curves for age less than ten years (long Rank Test).

The median follow-up for living patients was 109 months (10–237 months). Five and ten-years overall survival was 90.3% and 89%, respectively (fig 1). Five and 10-years EFS was 88.5% and 87%, respectively (fig 2). The ten years overall survival for clinical stage was: I (94.7%), II (91.3%), III (82.6%), and IV (71%) (p = 0.005). figure 6

### Prognostic factors

**Figure 4:**
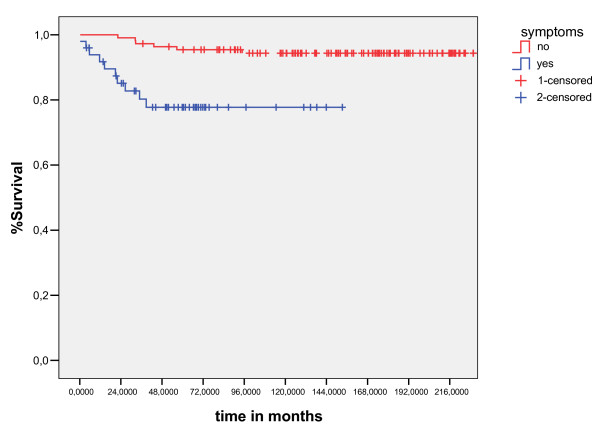
overall survival curves for B symptoms presence (log- Rank Test).

**Figure 5:**
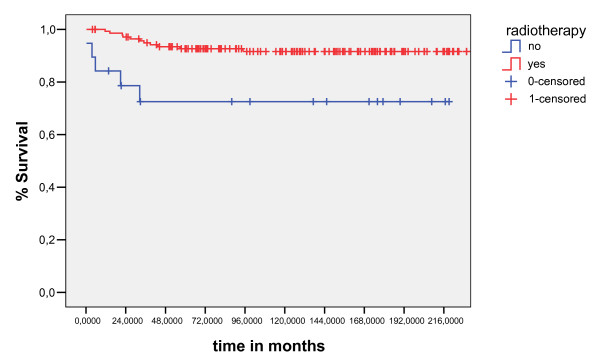
overall survival curves for No Radiotherapy (log- Rank Test).

**Figure 7:**
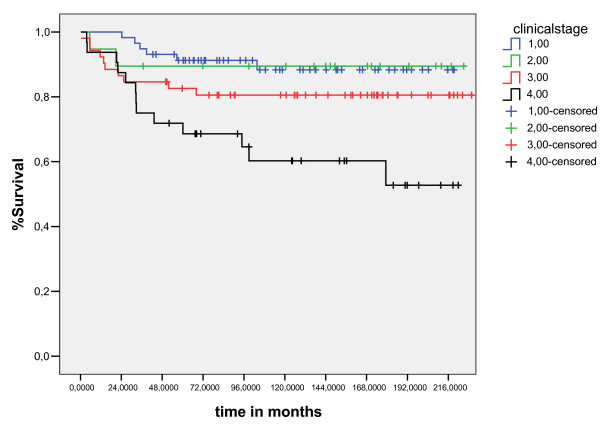
overall survival curves for clinical stage (log – Rank Test).

The results of the univariate analyses of the prognostic factors for EFS and OS are shown in Table 3. The significant prognostic factors associated unfavorable OS were: Age less than ten years(p = 0.01), no radiotherapy(p = 0.003), presence B symptoms (p = 0.0001) and clinical stage(p = 0.005)(figure 4, 5, 6, 7). Multivariate analysis revealed independent unfavorable prognostic factors for OS: advanced clinical stage (p = 0.02, HR = 9.2, IC95%-1.3 – 4.3), no radiotherapy (p = 0.03, HR = 4.3, IC95% – 1.2–9), and presence B symptoms. (p = 0.001, HR = 11, IC95% – 2–16.8) in table-5. The overall survival did not differ for patients treated with radiation therapy dose major than 21Gy (p = 0.75), sex (p = 0.6), mediastinuminvolvement (p = 0.66) or the schedule of chemotherapy (p = 0.27) done in the period of study did not influenced survival, as demonstrated table-4

**Table 4:** Univariate analysis of prognostic factors for Os and EFS*

**VARIABLE**							
**Age**	**number**	**%**	**Five years**	**OS %**	**EFS%**	**OS p**	**EFS p**
> 10	56	33.8		97.8	94.2	0.01	0.06
< 10	110	66.2		86.6	89.4		
**Sex**							
Female	116	69.8		89.4	96	0.84	0.6
Male	50	30.2		92.9	87		
**Histology**							
Ns	80	48.1		93	95	0.08	0.21
Mc	57	34.3		86.4	90		
Lp	26	15.6		98	80.4		
Dl	3	1.8		88.6	87		
**Stage**							
I	19	11.7		94.5	93	0.005	0.02
II	60	36.4		91	91		
III	55	32.1		82.5	80		
IV	32	19.8		71	69		
**Radiotherapy**							
Yes	146	87.9		91	94	0.003	0.07
No	20	12.1		72	88		
**RT dose**							
= < 21 GY	112	79.6		92.5	88.4	0.76	0.5
>21 Gy	34	20.4		89.1	90.2		
**Chemotherapy**							
ABVD	66	40,9		90.4	91	0.16	0.15
MOOP	22	13,6		92.6	88		
VEEP	17	10,5		90.7	91.2		
OTHERS	56	34,7		88.8	86		
**Symptoms B**							
Present	114	68.7		77.4	69.5	0.0001	0.0001
Absent	52	31.3		97.3	96.4		
**Mediast involved**							
Present	16	9.7		90	89	0.7	0.5
Absent	150	90.3		92.9	91		

* Kaplan Meier and log – Rank Test

**Table 5:** multivariate analyses of unfavourable significant factors for OS*

**Variable**	**p**	**HR**	**95%**	**confidential interval**
Age > 10 years	0.06	3.3	0.9	18.3
Present B Symptoms	0.001	11	2.07	16.8
No Radiotherapy	0.03	4.3	1.2	9
Advanced Clinical stage	0.02	9.2	1.3	4.3

*Cox Regression estimate, HR = hazard Risk

### Outcome in unfavorable risk subgroups

**Figure 3:**
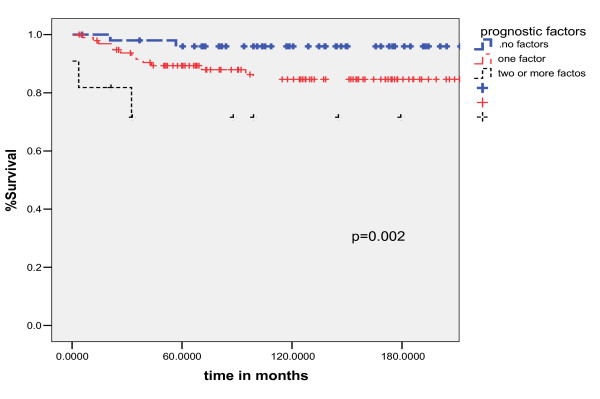
overall survival curves of unfavorable prognostic factors (log-Rank Test).

Using the three unfavorable independent prognostic factors for OS, the patients were divided into three risk groups to calculate the OS at five years (Fig. 3). For patients with no adverse prognostic factors (n = 52) the OS was 96%, for patients with one adverse prognostic factors (n = 98) the OS was 84.6% and for patients with two or more adverse prognostic factors (n = 11) OS was 71.6% (p = 0.002). The curves of overall survival for unfavorable prognostic factors are shown in figure 3.

### Second tumors

The incidence of second tumors was 1.8% (three cases) in fifteen years of follow-up. The cases of second tumors included two cases of thyroid carcinoma and one case of breast cancer. All cases were treated conformed protocols by cancer site.

## Discussion

Treatment of Hodgkin's disease usually combines chemotherapy (CT) and low dose radiation (RT) to obtain best results [14-23]. The rationale for this procedure is that while chemotherapy eradicates disseminated subclinical disease and small burden nodal disease, radiotherapy is considered necessary to improve local control and enhance duration of response of bulky lymphomas. The combination of both treatment modalities allows one to reduce the intensity and duration of chemotherapy as well as the dose and volume of RT. Early stage disease (I to IIA) presents excellent results with either combined treatment or chemotherapy alone, with DFS or EFS in excess of 90% [24]. In this cohort ten year OS and EFS was 94.5% and 93%, respectively, for clinical stage I and II. These data are compatible with other published results. The German-Austrian group carried out three studies with different RT doses schedules. In the HD90 study 25, 25, and 20 Gy was used in stages I-IIA, IIB-IIIA, and IIIB-IV, respectively, depending on the duration of chemotherapy; EFS among patients with localized disease was 92% to 96% [24]. The role of additional RT in stage III or IV disease remains controversial. Advanced disease (III to IV) requires more aggressive therapy. Subsequent trials from international study groups have obtained higher overall and disease-free survival rates, by increasing the dose intensity of treatment. Contemporary CMT trials report 5-year EFS rates greater than 80% in advanced disease. Chemotherapy and RT to bulky or residual disease provide a 10-year progression-free survival (PFS) or EFS of only 38% to 49% in stage IV [25,26]. Hybrid regimens using ABVD/ABV alternating with MOPP/COPP provide disease control in about 75% of stage IIB-IV patients without RT [27] but stage IV patients perform poorly with such conventional therapy, even if additional IFRT is administered: In our study patients with advanced stage IV obtained satisfactory OS and EFS in ten years 71 % and 69%, respectively. Our data suggest that advanced stage IV requires more aggressive therapy and radiotherapy has improved overall survival and event free survival rates. In the last decade, two major pediatric trials [28,29] have evaluated the utility of Low dose -IFRT in the treatment of Hodgkin's lymphoma. A trial of the former Children's Cancer Group (CCG) for children and adolescents with Hodgkin's lymphoma compared outcome in patients who achieved an initial complete response with chemotherapy followed by Low dose-IFRT or no further therapy. Complete response was defined as an absence of residual tumor or residual tumor that showed a reduction in size of 70% or more since diagnosis and a change from gallium positivity [28] Patients received risk-adapted chemotherapy (stages I-III, COPP/ABV; stage IV, more intensive therapy). The EFS for the 829 eligible patients was 85% at 5 years. Complete response was obtained in 83% of patients. Five hundred and one patients were randomized to receive low dose -IFRT or no further therapy. In an as-treated analysis, 3-year EFS was 93% for patients receiving Low dose -IFRT, and 85% for patients receiving no further therapy. Three-year survival for patients treated with and without low dose -IFRT was 98% and 99%, respectively [28]. The German Pediatric Oncology and Hematology Group (GPOH) initiated a study to assess the effect on EFS and OS of eliminating radiation for all patients achieving complete resolution of disease following chemotherapy [29]. Overall EFS was 92% for patients receiving radiation and 88% for those receiving no radiation (*P *= .05). In both the German GPOH-95 and CCG-5942 studies, the benefit of radiation therapy on EFS was greater in patients with advanced-stage disease at presentation.

In our institution in the period analyzed (1985–2000), IFRT was defined as treatment volume including the initially involved lymph node region(s) and 82.4% of initial clinical stage(I-II) patients in this series was treated with this volume of radiotherapy. Field definition for radiation therapy in unfavorable, and advanced Hodgkin's lymphoma was variable and protocol dependent. Although IFRT remained the standard when patients were treated with combined modality therapy, restricting radiation therapy to areas of initial bulk disease was possible to be used in 15% of the cases (stage III and IV) and in other cases mantle and extend fields were used mainly in stage III and IV (85%).

Protocols with MOPP chemotherapy (or derivatives) alone use higher cumulative doses of alkylating agents than CMT, and are associated with an increased risk of infertility and a higher cumulative risk of leukemia (7.9%) at 15 years after chemotherapy alone than after CMT (3.4%), as shown by the Late Effects Study Group [29,30]. The incidence in this cohort of second tumors was 1.8% (three cases) in fifteen years of follow-up. The cases of second tumors included two cases of thyroid carcinoma, one case of breast cancer and no cases of leukemia were seen. This data could be explained by the small number of patients who used high cumulative doses of MOOP and etoposide (23%). But indeed, long term follow-up of patients treated for HD in childhood shows a 18.5-fold increased risk of developing a second malignant neoplasm, mainly radiation-associated solid tumors (breast and thyroid cancers)[31].

Most publications in childhood HD have identified various prognostic factors. In the German Pediatric Oncology and Hematology Group (GPOH) GPOH-95 study, B symptoms, histology, and male sex were adverse prognostic factors for event-free survival on multivariate analysis [29]. In 320 children with clinically staged Hodgkin's lymphoma treated in the Stanford-St. Jude-Dana Farber Cancer Institute consortium, male gender; stage IIB, IIIB, or IV disease; white blood cell count 11,500/mm^3 ^or higher; and hemoglobin lower than 11.0 g/dL were significant on multivariate analysis for inferior disease-free survival and overall survival. Prognosis was associated with the number of adverse factors [32]. In the CCG-5942 study, the combination of B symptoms and bulky disease was associated with an inferior outcome [28,31,33,34]. In our analysis radiotherapy administration, early clinical stage, no B symptoms, were independent associated with improved overall survival. The prognostic factors associated with improved EFS were female gender, clinical stage, absent B symptoms and age less than ten years. These factors revealed that more efforts are still required in order to improve long-term survival in unfavorable and advanced disease as well as relapsed cases. Radiotherapy has a potential role in advanced Hodgkin's lymphoma reducing relapses in initially involved sites and improving survival.

## Conclusion

This data demonstrating the importance of RT consolidation with low dose and reduced volume, in all clinical stage of childhood HD, producing satisfactory ten years OS and EFS. Our results, though retrospective, suggests that patients who were able to receive RT performed better and were expected to achieve better local control when compared to patients whom for any reason were not able to undergo RT. As the disease is highly curable, any data of long term follow-up should be presented in order to better direct therapy, and to identify groups of patients who would not benefit from radiation treatment.
